# Genotype-phenotype association and biochemical analyses of glucose-6-phosphate dehydrogenase variants: Implications for the hemolytic risk of using 8-aminoquinolines for radical cure

**DOI:** 10.3389/fphar.2022.1032938

**Published:** 2022-10-20

**Authors:** Sirapapha Sudsumrit, Kamonwan Chamchoy, Duantida Songdej, Poom Adisakwattana, Srivicha Krudsood, Emily R. Adams, Mallika Imwong, Ubolsree Leartsakulpanich, Usa Boonyuen

**Affiliations:** ^1^ Department of Molecular Tropical Medicine and Genetics, Faculty of Tropical Medicine, Mahidol University, Bangkok, Thailand; ^2^ Princess Srisavangavadhana College of Medicine, Chulabhorn Royal Academy, Bangkok, Thailand; ^3^ Department of Pediatrics, Faculty of Medicine Ramathibodi Hospital, Mahidol University, Bangkok, Thailand; ^4^ Department of Helminthology, Faculty of Tropical Medicine, Mahidol University, Bangkok, Thailand; ^5^ Department of Tropical Hygiene, Faculty of Tropical Medicine, Mahidol University, Bangkok, Thailand; ^6^ Research Centre for Drugs and Diagnostics, Liverpool School of Tropical Medicine, Liverpool, United Kingdom; ^7^ National Center for Genetic Engineering and Biotechnology, National Science and Technology Development Agency, Pathumthani, Thailand

**Keywords:** G6PD deficiency, genotype, phenotype, 8-amioquinolines, hemolytic risk

## Abstract

**Background:** *Plasmodium vivax* remains the malaria species posing a major threat to human health worldwide owing to its relapse mechanism. Currently, the only drugs of choice for radical cure are the 8-aminoquinolines (primaquine and tafenoquine), which are capable of killing hypnozoites and thus preventing *P. vivax* relapse. However, the therapeutic use of primaquine and tafenoquine is restricted because these drugs can cause hemolysis in individuals with glucose-6-phosphate dehydrogenase (G6PD) deficiency. This study aimed to assess and understand the hemolytic risk of using 8-aminoquinolines for radical treatment in a malaria endemic area of Thailand.

**Methods:** The prevalence of G6PD deficiency was determined using a quantitative test in 1,125 individuals. Multiplexed high-resolution meltinging (HRM) assays were developed and applied to detect 12 G6PD mutations. Furthermore, biochemical and structural characterization of G6PD variants was carried out to understand the molecular basis of enzyme deficiency.

**Results:** The prevalence of G6PD deficiency was 6.76% (76/1,125), as assessed by a phenotypic test. Multiplexed HRM assays revealed G6PD Mahidol in 15.04% (77/512) of males and 28.38% (174/613) of females, as well as G6PD Aures in one female. G6PD activity above the 30% cut-off was detected in those carrying G6PD Mahidol, even in hemizygous male individuals. Two variants, G6PD Murcia Oristano and G6PD Songklanagarind + Viangchan, were identified for the first time in Thailand. Biochemical characterization revealed that structural instability is the primary cause of enzyme deficiency in G6PD Aures, G6PD Murcia Oristano, G6PD Songklanagarind + Viangchan, and G6PD Chinese 4 + Viangchan, with double G6PD mutations causing more severe enzyme deficiency.

**Conclusion:** In western Thailand, up to 22% of people may be ineligible for radical cure. Routine qualitative tests may be insufficient for G6PD testing, so quantitative tests should be implemented. G6PD genotyping should also be used to confirm G6PD status, especially in female individuals suspected of having G6PD deficiency. People with double G6PD mutations are more likely to have hemolysis than are those with single G6PD mutations because the double mutations significantly reduce the catalytic activity as well as the structural stability of the protein.

## 1 Introduction

Despite a downward trend in infection rates over the years, the World Health Organization (WHO) reported approximately 241 million malaria cases in 2020, with 765,000 deaths (Organization, 2021). *P. vivax* remains the predominant species in many areas, including Thailand. Vivax malaria has a relapse mechanism caused by the dormant liver stage (hypnozoite), resulting in the parasites reappearing months or even years after initial infection (Krotoski et al., 1982; White, 2011). Consequently, *P. vivax* is responsible for the high worldwide morbidity rate for malaria (Guerra et al., 2010; Organization, 2021). The drugs of choice for radical treatment of malaria are 8-aminoquinolines (primaquine and tafenoquine), and they play a critical role in the elimination and control of *P. vivax* (Alving et al., 1955; Llanos-Cuentas et al., 2019; Watson and Taylor, 2019). They are the only medications capable of preventing relapse by killing the hypnozoites of *P. vivax*. However, there are safety concerns regarding the use of these drugs in people who have X-linked glucose-6-phosphate dehydrogenase (G6PD) deficiency (Beutler et al., 1955; Rueangweerayut et al., 2017). G6PD is an enzyme that catalyzes the formation of reduced nicotinamide adenine dinucleotide phosphate (NADPH), a cellular reducing power. G6PD is especially important in non-nucleated red blood cells (RBCs) because it is the only NADPH producer in these cells. Hemolysis can occur under oxidative stress conditions in people with G6PD deficiency owing to insufficient NADPH levels. Radical treatment using 8-amioquinolines was found to be associated with acute hemolysis in G6PD-deficient individuals (Beutler et al., 1955; Rueangweerayut et al., 2017; Chu et al., 2018). Furthermore, G6PD deficiency is common in malaria-endemic areas (Africa, America and Southeast Asia), potentially limiting the use of primaquine and tafenoquine for radical treatment (Nkhoma et al., 2009; Howes et al., 2012; Recht et al., 2018).

The hemolytic toxicity of 8-aminoquinolines is determined by two major factors: oxidative stress exposure (exposure time and level of oxidative stress) and the G6PD enzyme activity level in RBCs. The level of oxidative stress is determined by the dose regimen, whereas G6PD enzyme activity is determined by the G6PD genotype. Drug-induced hemolysis in G6PD deficiency was found to be dose-dependent for 8-aminoquinolines (Chu et al., 2017; Rueangweerayut et al., 2017; Chu et al., 2018). The standard dose of primaquine is 15 mg/day for 14 days, whereas tafenoquine is generally prescribed as a single 300-mg dose. However, tafenoquine administration is recommended only for individuals with at least 70% G6PD activity (Lacerda et al., 2019; Commons et al., 2020). The activity level of the G6PD enzyme is affected by the G6PD mutation type. G6PD variants are classified into five groups, on the basis of enzyme activity level and clinical manifestations (Yoshida et al., 1971). The most severe enzyme deficiency is caused by class I variants, whereas class V variants have increased enzyme activity without clinical outcomes.

Because of the adverse events that can be caused by 8-aminoquinolines, G6PD testing is recommended by WHO when available before radical cure (Organization, 2016a). G6PD activity is routinely measured in phenotypic tests by quantitative or qualitative methods. The standard quantitative method is spectrophotometry, which measures NADPH production at 340 nm. Qualitative G6PD tests, such as the fluorescent spot test and the CareStart™ G6PD rapid diagnostic test give a deficient or normal read out for G6PD deficiency. Currently available tests reliably identify samples with G6PD activity ≤30% of normal. Hence, qualitative methods can correctly identify hemizygous male individuals and homozygous female individuals. However, studies have found that heterozygous female individuals are incorrectly classified as G6PD normal by routine qualitative tests (Chu et al., 2017; Islam et al., 2018). Female individuals who are heterozygous for G6PD deficiency exhibit a wide range of G6PD activity levels because they have mixed populations of normal and G6PD-deficient RBCs due to random X-inactivation during embryonic development (Harper, 2011). G6PD genotyping is not widely used because the necessary procedures are complex and expensive. Nonetheless, genotypic testing could be useful as a supplement to identify heterozygous female individuals with G6PD deficiency.

Between October 2021 and June 2022, Thailand reported 4,764 malaria cases. There were 3,610 malaria cases in the western region, accounting for 76% of all cases, among which *P. vivax* was the most common causative species with a 97% incidence rate (Malaria situation in Thailand, 2022). These data highlight the importance of using 8-aminoquinolines in this region. Previous studies showed a high prevalence of G6PD deficiency in people living along the Thai‒Myanmar border (Phompradit et al., 2011; Bancone et al., 2014; Bancone et al., 2017a; Boonyuen et al., 2021). However, owing to the small sample size and nature of the qualitative tests used in those studies, information on the prevalence of G6PD deficiency in the western region is still limited. Furthermore, data on G6PD genotype are still scarce because G6PD genotyping has not been widely performed in this region. Indeed, information on the prevalence and genotype of G6PD deficiency is critical for estimating the percentage of eligible individuals and assessing the risk of hemolysis in the population, to allow for the safe and effective use of 8-aminoquinolines. Knowledge on the hemolytic risk of a specific variant is also of importance to guide the use of primaquine and tafenoquine. In addition to clinical data, biochemical characterization, which provides information such as binding affinity, catalytic efficiency, and thermostability, is critical because it explains how each specific variant operates and affects enzyme activity, which reflects the response to oxidative stress in the RBCs of individuals with each variant.

The purpose of this study was to determine the prevalence and genotypes of G6PD deficiency in western Thailand, a malaria endemic area, for the purpose of assessing and understanding the hemolytic risk of using 8-aminoquinolines for radical cure of *P. vivax*. The prevalence of G6PD deficiency was examined in 1,125 people by using a quantitative G6PD activity assay based on water-soluble tetrazolium salts (WST-8). G6PD genotyping was performed using a developed multiplexed high-resolution melting (HRM) analysis that can detect 12 G6PD mutations common in Southeast Asia, allowing for high-throughput G6PD genotyping. Furthermore, to provide insight into the molecular mechanisms underlying enzyme deficiency of G6PD variants, the biochemical and structural characteristics of four G6PD mutations (G6PD Aures, G6PD Murcia Oristano, G6PD Songklanagarind + Viangchan, and G6PD Chinese 4 + Viangchan) were described.

## 2 Materials and methods

### 2.1 Blood samples

A total of 1,125 blood samples were collected in ethylenediaminetetraacetic acid (EDTA) tubes and stored at −20°C until use. The participants were healthy residents living in western Thailand, along the Thai-Myanmar border. Malaria status and anemia were not tested before recruitment. For the validation of HRM assays, blood samples were collected from healthy Thai volunteers at the Faculty of Medicine Ramathibodi Hospital, Bangkok, Thailand. The integrity of samples for phenotypic analysis was maintained under these storage conditions (Chamchoy et al., 2021).

### 2.2 Phenotypic screening for glucose-6-phosphate dehydrogenase deficiency by enzyme activity assay (water-soluble tetrazolium salts)

The blood was mixed with the reaction mixtures containing 20 mM Tris-HCl pH 8.0, 10 mM MgCl_2_, 500 μM glucose-6-phosphate (G6P), 100 μM NADP^+^, and 100 μM WST-8 (Sigma-Aldrich, Darmstadt, Germany). The enzymatic reaction was monitored by following the absorbance at 450 nm using a microplate reader (Sunrise; Tecan, Männedorf, Switzerland). A reaction mixture set up in the absence of substrates was used for background subtraction. Hemoglobin concentration was measured using Drabkin’s reagent (Sigma-Aldrich), following the manufacturer’s instructions. The G6PD enzyme activity was expressed as units (U) per gram of hemoglobin (gHb).

### 2.3 DNA extraction

DNA extraction was performed using the QIAamp DNA Blood Mini Kits (QIAGEN, Hilden, Germany), following the manufacturer’s instructions. For DNA extraction, 100 μl blood sample was used and eluted into a final volume of 100 μl. DNA concentration was measured using a NanoDrop 2000 spectrophotometer (Thermo Fisher Scientific, Waltham, MA, United States).

### 2.4 Glucose-6-phosphate dehydrogenase genotyping using multiplexed high-resolution melting

**FIGURE 1:**
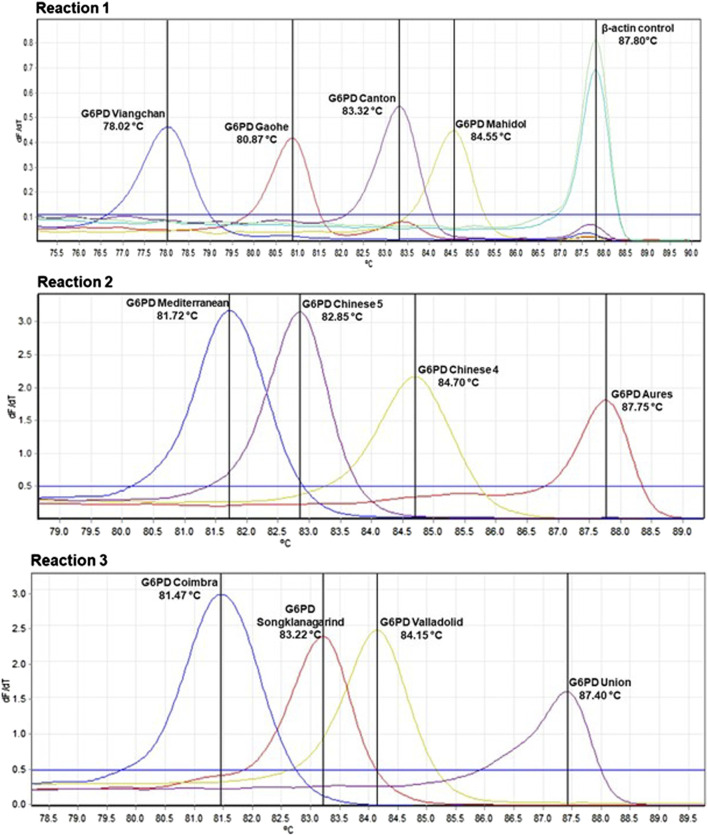
Detection of G6PD mutations by multiplexed HRM assays. Samples with G6PD mutations produce a peak at corresponding melting temperature (Tm). *β*-Actin amplification, which was included in reaction 1, was used to confirm the integrity of the gDNA template.

In our previous study, we developed multiplexed HRM assays to detect eight G6PD variants that are common in Thailand and Southeast Asia (Boonyuen et al., 2021). We modified and expanded those assays in this study to detect a total of 12 G6PD mutations in three reactions. The primers (Table 1) were designed to detect mutations by producing PCR products with different melting temperatures (*T*_*m*_). To maximize the sensitivity and specificity of the assay, several assay conditions, including primer concentrations, assay protocol, and detection conditions were optimized (Table 2; Figure 1). A total volume of 12.5 μl was used for the multiplexed HRM assays, which included 6.25 μl of 1 × HRM Type-It mix (QIAGEN), different concentrations of each primer, molecular-grade water, and 2.5 μl of the gDNA template. PCR amplification and a melting curve analysis were carried out with the Rotor-Gene Q (QIAGEN) under the following conditions: one cycle at 95°C for 5 min, followed by 30 cycles at 95°C for 10 s, 63°C for 30 s, and 72°C for 10 s. Following that, an HRM analysis was carried out by melting from 75°C–90°C and taking readings at every 0.1°C step with 2 s of stabilization. Every run included both positive (gDNA with known mutations, confirmed by DNA sequencing) and negative [gDNA of G6PD wild-type (WT), confirmed by DNA sequencing] controls. In addition, *β-*Actin amplification was included in reaction 1 as an internal control to ensure the integrity of the gDNA. The Rotor-Gene Q software was used to analyze the data.

**TABLE 1:** Primers used in multiplexed HRM assays.

Primer	G6PD variant	Amino acid change	Primer sequence
A95G_F	Gaohe	His32Arg	5′- TTC CAT CAG TCG GAT ACA CG -3′
A95G_R			5′- AGG CAT GGA GCA GGC ACT TC -3′
T143C_F	Aures	Ile48Thr	5′- GAC CTG GCC AAG AAG AAG AC -3′
T143C_R			5′- CCG GCC ATC CCG GAA CAG CC -3′
T196A_F	Songklanagarind	Phe66Ile	5′- CCT TCT GCC CGA AAA CAC CA -3′
T196A_R			5′- AAG GGC TCA CTC TGT TTG CG -3′
G392T_F	Chinese 4	Gly131Val	5′- CAT GAA TGC CCT CCA CCT GT -3′
G392T_R			5′- TTC TTG GTG ACG GCC TCG TA -3′
C406T_F	Valladolid	Arg136Cys	5′- CCT GGG GTC ACA GGC CAA CT -3′
C406T_R			5′- CTC ATG CAG GAC TCG TGA AT -3′
G487A_F	Mahidol	Gly163Ser	5′- TCC GGG CTC CCA GCA GAA -3′
G487A_R			5′- GGT TGG ACA GCC GGT CA -3′
C563T_F	Mediterranean	Ser188Phe	5′- CGG CTG TCC AAC CAC ATC TT -3′
C563T_R			5′- GTT CTG CAC CAT CTC CTT GC -3′
C592T_F	Coimbra	Arg198Cys	5′- CCG TGA GGA CCA GAT CTA CT -3′
C592T_R			5′- CCC CAC CTC AGC ACC ATG -3′
G871A_F	Viangchan	Val291Met	5′- GGC TTT CTC TCA GGT CAA GA-3′
G871A_R			5′- CCC AGG ACC ACA TTG TTG GC-3′
C1024T_F	Chinese 5	Leu342Phe	5′- CAC TTT TGC AGC CGT CGT CT-3′
C1024T_R			5′- CAC ACA GGG CAT GCC CAG TT-3′
C1360T_F	Union	Arg454Cys	5′- GAG CCA GAT GCA CTT CGT GT -3′
C1360T_R			5′- GAG GGG ACA TAG TAT GGC TT -3′
G1376T_F	Canton	Arg459Leu	5′- CCT CAG CGA CGA GCT CCT -3′
G1376T_R			5′- CTG CCA TAA ATA TAG GGG ATG G -3′

**TABLE 2:** Multiplexed HRM conditions.

Reaction	Variant	Primer	Primer concentration (nM)	*T*_*m*_ of PCR product (°C)	Product size (bp)
1	G6PD Gaohe	A95G_F	600	80.87	100
		A95G_R			
	G6PD Mahidol	G487A_F	400	84.55	87
		G487A_R			
	G6PD Viangchan	G871A_F	600	78.02	66
		G871A_R			
	G6PD Canton	G1376T_F	600	83.32	99
		G1376T_R			
2	G6PD Aures	T143C_F	400	87.75	152
		T143C_R			
	G6PD Chinese 4	G392T_F	200	84.70	87
		G392T_R			
	G6PD Mediterranean	C563T_F	400	81.72	87
		C563T_R			
	G6PD Chinese 5	C1024T_F	400	82.85	99
		C1024T_R			
3	G6PD Songklanagarind	T196A_F	400	83.22	84
		T196A_R			
	G6PD Valladolid	C406T_F	400	84.15	93
		C406T_R			
	G6PD Coimbra	C592T_F	400	81.47	78
		C592T_R			
	G6PD Union	C1360T_F	200	87.40	127
		C1360T_R			

For the genotypic test, direct DNA sequencing was used as a reference test. HRM assays were validated using 100-normal and 100-deficient G6PD samples. Genomic DNA was extracted and the *G6PD* gene was amplified using previously described conditions (Boonyuen et al., 2021). PCR products were subjected to purification and sequenced (first BASE, Apical Scientific, Malaysia).

### 2.5 Biochemical and structural characterization of glucose-6-phosphate dehydrogenase variants

To investigate the molecular mechanisms underlying enzyme deficiency of G6PD variants, biochemical and structural characterization were carried out. G6PD mutations were created by site-directed mutagenesis using pET28a-G6PD WT as a template and the presence of desired mutations was confirmed by DNA sequencing. Primers used for site-directed mutation are listed in Table 3. The PCR conditions for site-directed mutagenesis were previously described (Pakparnich et al., 2021). G6PD protein expression was performed using *E. coli* BL21 (DE3). The bacteria were cultured at 37°C with 250 rpm shaking in the presence of 50 μg/ml kanamycin until the OD_600_ reached one and the G6PD expression was induced with 1 mM IPTG. Cells were then cultured for 20 h at 20°C with 200 rpm shaking before being harvested by centrifugation.

**TABLE 3:** Primers used for site-directed mutagenesis.

Nucleotide change	Primer	Primer sequence
T143C	Aures_F	5′-AGA AGA AGA CCT ACC CCA CC -3′
	Aures_R	5′-GGT GGG GTA GGT CTT CTT CT -3′
T196A	Songklanagarind_F	5′- CCC GAA AAC ACC ATC ATC GTG GGC -3′
	Songklanagarind_R	5′- GCC CAC GAT GAT GGT GTT TTC GGG -3′
A209G	Murcia Oristano_F	5′-TCG TGG GCT GTG CCC GTT CC -3′
	Murcia Oristano_R	5′- GGA ACG GGC ACA GCC CAC GA -3′
G392T	Chinese 4_F	5′-CCT CCA CCT GGT GTC ACA G-3′
	Chinese 4_R	5′-CTG TGA CAC CAG GTG GAG G-3′
G871A	Viangchan_F	5′-GAT GAG AAG GTC AAG ATG TTG AAA TG -3′
	Viangchan_R	5′-GAT GCA TTT CAA CAT CTT GAC CTT CTC-3′

Protein purification was carried out using immobilized metal affinity chromatography in accordance with the previously described protocols (Boonyuen et al., 2017). Protein purity was visualized with SDS-PAGE and the Bradford assay was used to determine protein concentration (Bradford, 1976).

To assess the effect of mutations on the catalytic activity of G6PD variants, steady state kinetic parameters were determined. The standard reaction contained 20 mM Tris-HCl pH 8.0, 10 mM MgCl_2_, 500 μM G6P, and 100 μM NADP^+^. The enzymatic reaction was monitored by following the formation of NADPH at 340 nm using UV-VIS spectrophotometer (Shimadzu, Kyoto, Japan). To determine the *K*_m_ for G6P, the concentration of NADP^+^ was fixed at 100 μM while varying the concentrations of G6P from 2.5 to 1,000 μM, and to determine the *K*_m_ for NADP^+^, the concentration of G6P was fixed at 500 μM while varying the concentrations of NADP^+^ from 1 to 200 µM.

To examine the effect of mutations on the secondary structure of G6PD variants, the secondary structure was analyzed using circular dichroism (CD). Far UV-CD spectra of the G6PD variants (0.1 mg/ml) were recorded in a 1-mm path-length quartz cuvette at 25°C using a Jasco spectrometer, model J-815, equipped with a Peltier temperature control system. The CD spectra were collected over a wavelength range of 190–260 nm at a scan rate of 50 nm/min. Five scans were averaged for each sample, and the results of the buffer scans were subtracted.

To investigate the effect of mutations on structural stability, each G6PD variant was subjected to thermal stability analysis and their susceptibility to guanidine hydrochloride (Gdn-HCl) treatment and trypsin digestion were determined. Thermal stability testing was performed in a 20-μl reaction, containing protein at a concentration of 0.25 mg/ml mixed with 5 × SYPRO™ Orange Protein Gel Stain (Thermo Fisher Scientific, San Jose, CA, United States). The reaction mixtures were heated in a Light- Cycler 480 real-time PCR machine (Roche, Mannheim, Germany) at temperatures ranging from 20°C to 80°C, with excitation and emission wavelengths of 465 and 580 nm, respectively. Furthermore, the effect of NADP^+^, which is known to stabilize the G6PD protein structure, was investigated by incubating the protein with various concentrations of NADP^+^ (0, 10, and 100 µM). The *T*_*m*_ of each G6PD variant was calculated and defined as the temperature at which half of the protein was unfolded.

To investigate the structural stability of G6PD variants upon chemical denaturation, the protein was treated with different concentrations of Gdn-HCl (0–0.5 M) in the presence of various concentrations of NADP^+^ (0, 10, and 100 µM) at 37°C for 2 h. The residual enzyme activity was measured and expressed as a percentage of the activity of the same enzyme incubated without Gdn-HCl.

Finally, to determine the susceptibility of G6PD variants to trypsin digestion, the protein was treated with trypsin (0.5 mg/ml) for 5 min at 25°C in the presence of various concentrations of NADP^+^ (0, 10, and 100 µM). The residual enzyme activity was measured and expressed as a percentage of the activity of the same enzyme incubated without trypsin.

## 3 Results

### 3.1 Screening of glucose-6-phosphate dehydrogenase deficiency by phenotypic water-soluble tetrazolium salts assay

**FIGURE 2:**
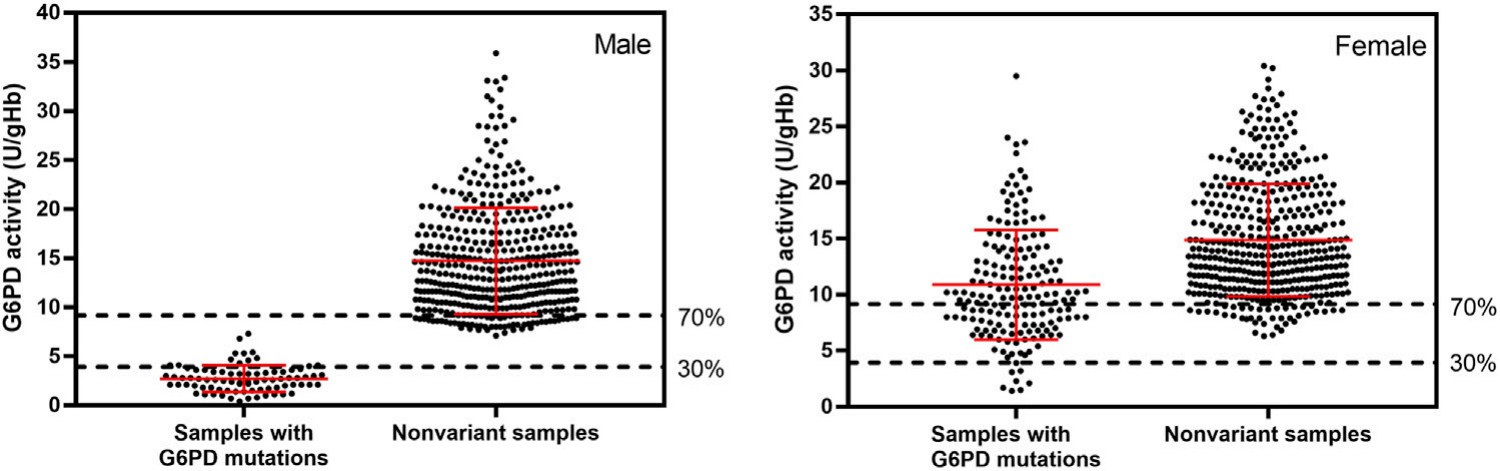
G6PD activity of 1,125 samples with and without G6PD mutations, measured by WST-8 assay. The adjusted male median for the studied population was 13.1 ± 6.5 U/gHb, which was defined as 100% G6PD activity. Average G6PD activity in male and female participants with detected mutations was 2.68 ± 1.37 and 11.04 ± 4.97 U/gHb, respectively. Nonvariant male and female participants had average G6PD activity values of 14.74 ± 5.79 and 14.89 ± 5.37 U/gHb, respectively. Mutations were identified by multiplexed HRM assays. The dotted horizontal lines represent AMM activity levels of 30% and 70%, which are considered G6PD deficient and intermediate, respectively.

Screening of G6PD deficiency was initially accomplished by performing a phenotypic enzyme activity assay (WST-8). The average G6PD activity among 1,125 samples was 13.32 ± 5.97 U/gHb, with male participants showing an average G6PD activity of 12.81 ± 6.65 U/gHb and female participants showing a slightly higher average G6PD activity of 13.74 ± 5.30 U/gHb. The adjusted male median (AMM) was determined to be 13.1 ± 6.5 U/gHb for the studied population and was defined as 100% G6PD activity (Domingo et al., 2013). According to WHO, a G6PD activity level of <30% of the AMM is considered to be deficient, and a G6PD activity level of between 30% and 80% of the AMM is considered to be intermediately deficient (Organization, 2016b). However, the administration of tafenoquine is recommended for only those with a G6PD activity of at least 70% (Llanos-Cuentas et al., 2019). Therefore, in this study, a G6PD activity level of <30% was considered to be deficient, while an activity level of between 30% and 70% was considered to be intermediate. According to the WST-8 assay results, the prevalence of G6PD deficiency among 1,125 study volunteers living in the western Thailand was 6.76% (76/1,125). By sex, the prevalence of G6PD deficiency was 13.28% (68/512) in male participants and 1.30% (8/613) in female participants. Intermediate G6PD activity was detected in 13.48% (69/512) of the male participants and in 16.48% (101/613) of the female participants (Figure 2).

**FIGURE 3:**
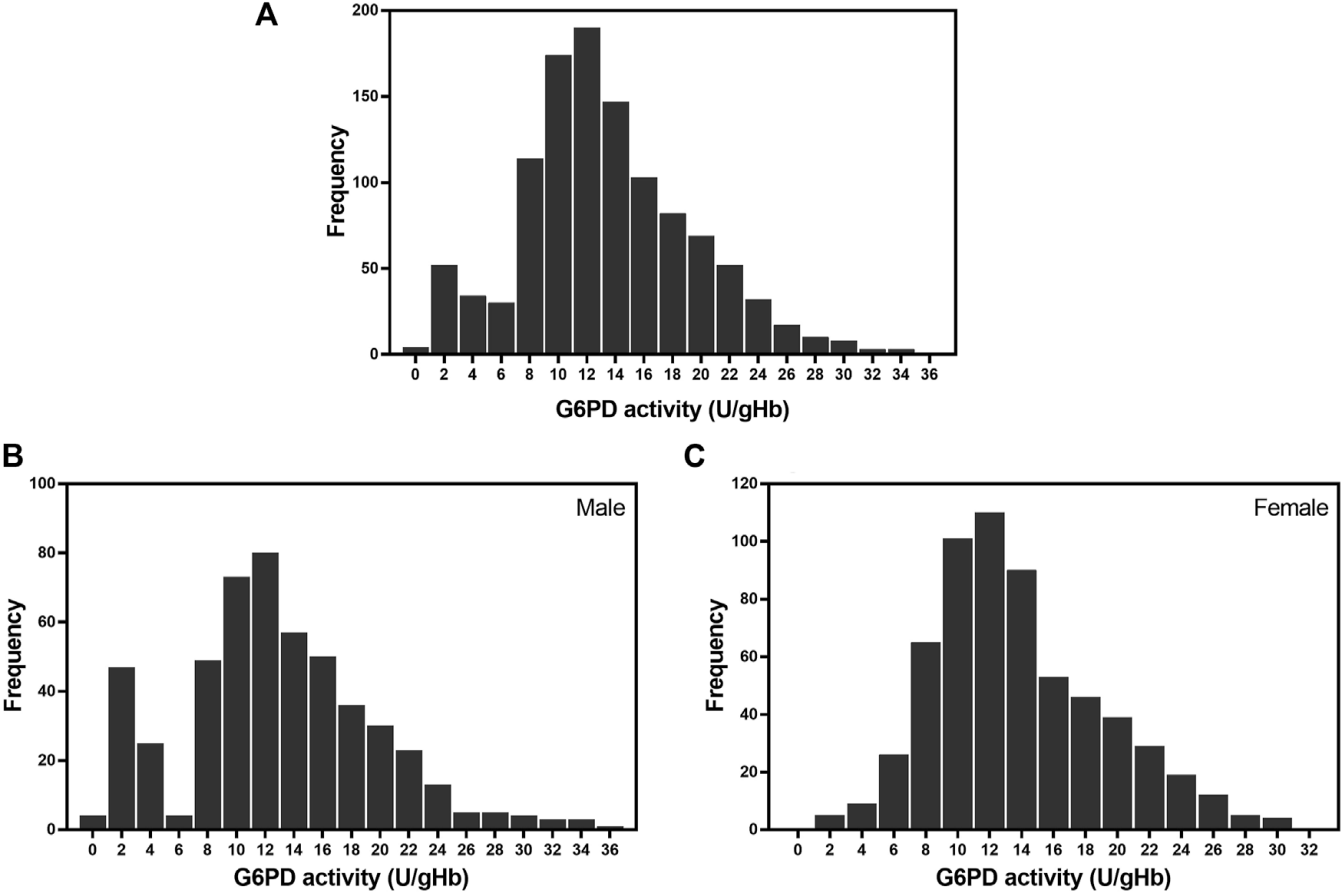
Frequency distribution of G6PD activity in the studied population. **(A)** Frequency of G6PD activity in 1,125 samples. The average G6PD activity in the studied population was 13.32 ± 5.97 U/gHb. **(B)** Frequency of G6PD activity in 512 male participants. The average G6PD activity in male participants was 12.81 ± 6.65 U/gHb. **(C)** Frequency of G6PD activity in 613 female participants. The average G6PD activity in female participants was 13.74 ± 5.30 U/gHb.

The frequency distribution of G6PD activity, as determined using the WST-8 assay, for the 1,125 samples is presented in Figure 3A. The majority of enzyme activity levels in the studied population were between 8 and 18 U/gHb. Figures 3B,C display the frequency distribution of G6PD activity in male and female participants, respectively. The frequency distribution of the male participants has two distinct peaks, indicating that there are two groups of G6PD status: deficient and normal. In contrast, the frequency distribution for the female participants has only one continuous peak.

### 3.2 Glucose-6-phosphate dehydrogenase genotyping by multiplexed high-resolution melting assays

Multiplexed HRM assays were designed to detect 12 mutations common in Southeast Asia over three reactions, with *β*-Actin serving as an internal control. Reaction 1 detects G6PD Gaohe (A95G), G6PD Mahidol (G487A), G6PD Viangchan (G871A) and G6PD Canton (G1376T); reaction 2 detects G6PD Aures (T143C), G6PD Chinese 4 (G392T), G6PD Mediterranean (C563T) and G6PD Chinese 5 (C1024T); and reaction 3 detects G6PD Songklanagarind (T196A), G6PD Valladolid (C406T), G6PD Coimbra (C592T) and G6PD Union (C1360T). The assays were validated with 100 normal and 100 G6PD-deficient samples, using DNA sequencing as a reference test. The HRM assays were 100% sensitive [95% confidence interval (CI): 96.30%–100%] and 100% specific (95% CI: 96.30%–100%) for detecting these 12 mutations. Among the 100 G6PD-deficient samples used for validation, three double mutations were detected which included G6PD Mahidol + Canton (G487A + G1376T), G6PD Chinese 4 + Viangchan (G392T + G871A) and G6PD Songklanagarind + Viangchan (T196A + G487A). In addition, G6PD Murcia Oristano (A209G) was discovered for the first time in G6PD-deficient samples from the Thai population, using DNA sequencing.

When multiplexed HRM assays were used to detect 12 mutations, we found that 22.40% (252/1,125) of the studied population carried a G6PD mutation. These mutations were detected in 15.04% (77/512) of the male participants and in 28.55% (175/613) of female participants. G6PD Mahidol was the predominant G6PD variant in the studied population, accounting for 99.60% of detected mutations (251/252). Another variant discovered in our study population was G6PD Aures, which was found in a heterozygous female participant with a G6PD enzyme activity level of 11.2 U/gHb. There were two male participants who had low enzyme activity (3.3 and 4.1 IU/gHb) but no G6PD mutation detected by our HRM assays. These two samples were amplified for G6PD and sent for DNA sequencing. It was revealed that both of them carried G6PD Kaiping (G1388A). The G6PD activity levels of samples with detected mutations and of samples with no detected mutations (nonvariant) are depicted in Figure 2. The average G6PD activity in male and female participants with detected mutations was 2.68 ± 1.37 and 11.04 ± 4.97 U/gHb, respectively. It is worth noting that female participants who carried G6PD mutations exhibited a wide range of G6PD activity levels, ranging from 1.4 to 29.5 U/gHb. In contrast, nonvariant male and female participants had comparable average G6PD activity values of 14.74 ± 5.79 and 14.89 ± 5.37 U/gHb, respectively.

**FIGURE 4:**
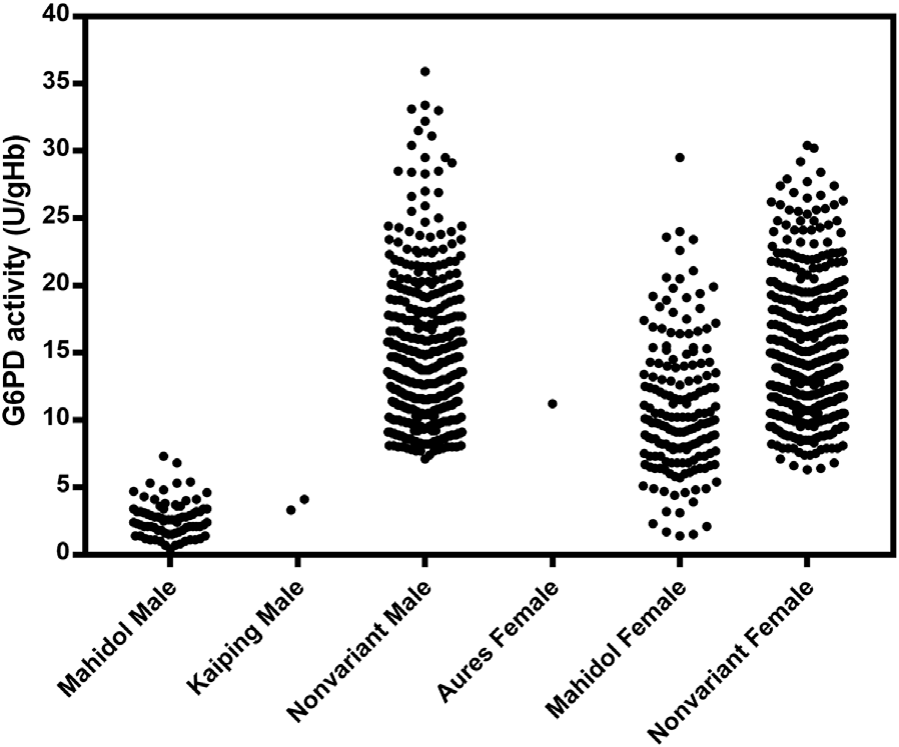
Distribution of G6PD activity by mutation type.

The distribution of G6PD activity levels by mutation type is presented in Figure 4. Hemizygous male participants with G6PD Mahidol showed low enzyme activity levels in the range of 0.4–7.3 U/gHb. Nonetheless, it should be noted that 30% of the AMM in this population was 3.93 U/gHb, implying that phenotypic tests with a 30% cut-off may be insufficient to accurately identify hemizygous G6PD-deficient male individuals. As expected, female participants with G6PD Mahidol showed a wide range of G6PD activity levels (1.4–29.5 U/gHb) because most of them were heterozygous for G6PD deficiency. Female participants carrying G6PD Mahidol had a slightly lower average G6PD activity level compared with those with no detected mutations; their average G6PD activity levels were 11.04 ± 4.97 U/gHb and 14.89 ± 5.37 U/gHb, respectively.

### 3.3 Biochemical and structural characterization of glucose-6-phosphate dehydrogenase variants

In the present study, one single variant (G6PD Murcia Oristano) was discovered for the first time in Thailand, as was a double variant (G6PD Songklanagarind + Viangchan). While many G6PD variants have been extensively studied, the biochemical properties of two single G6PD variants (G6PD Aures and G6PD Murcia Oristano) and two double G6PD variants (G6PD Chinese 4 + Viangchan and G6PD Songklanagarind + Viangchan) have never been reported. Therefore, biochemical and structural characterizations were carried out on these G6PD variants to better understand the molecular mechanisms underlying enzyme deficiency.

#### 3.3.1 Biochemical properties of glucose-6-phosphate dehydrogenase variants

Four G6PD variants were purified to homogeneity using immobilized metal affinity chromatography (Supplementary Material) and their biochemical and structural properties were investigated. Steady state kinetic parameters were determined (Table 4). The catalytic activity of G6PD variants was only moderately affected by the single G6PD mutations (G6PD Aures and G6PD Murcia Oristano), whereas a significant effect on the catalytic activity was observed for the double G6PD mutations. The G6PD mutations have a negligible effect on binding affinity toward either substrates (G6P or NADP^+^). In terms of catalytic efficiency (*k*_*cat*_/*K*_m_), the Class III single variants exhibited approximately 2‒ and 3‒fold lessened amounts of G6P and NADP^+^ catalysis, respectively. The double G6PD variants, in contrast, had significantly lower catalytic efficiencies. The catalytic efficiency toward G6P and NADP^+^ catalysis was approximately 22‒ and 46‒fold lower for G6PD Songklanagarind + Viangchan, respectively, and was 27‒ and 40‒fold lower, respectively, for G6PD Chinese 4 + Viangchan, when compared with that for the G6PD WT.

**TABLE 4:** Steady-state kinetic parameters of G6PD variants.

Construct	Class	Amino acid change	*k*_cat_ (s^−1^)	*K*_m_ G6P (µM)	*K*_m_ NADP^+^ (µM)
WT	**—**	**—**	206 ± 5	45 ± 6	9 ± 1
Aures	III	Ile48Thr	85 ± 4	34 ± 3	11 ± 4
Murcia Oristano	III	Tyr70Cys	156 ± 8	52 ± 7	19 ± 4
Songklanagarind + Viangchan	NR	Phe66Ile + Val291Met	9 ± 1	44 ± 2	18 ± 4
Chinese 4 + Viangchan	NR	Gly131Val + Val291Met	11 ± 2	64 ± 10	19 ± 5

#### 3.3.2 Structural stability of glucose-6-phosphate dehydrogenase variants

##### 3.3.2.1 Secondary structure analysis

**FIGURE 5:**
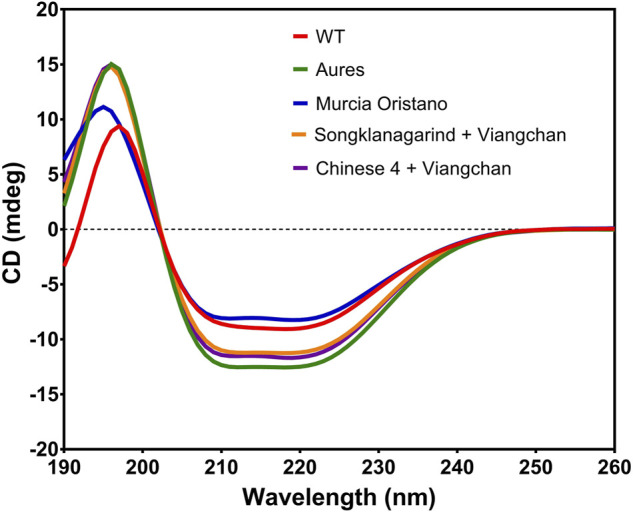
Secondary structure analysis of G6PD variants by CD. A Jasco spectrometer, model J-815, was used to collect CD spectra at 25°C over a wavelength range of 190–260 nm at a scan rate of 50 nm/min.

The effect of mutations on the structural stability of G6PD variants was investigated. First, the effect of mutations on the secondary structure was evaluated by recording the CD spectra of four G6PD variants in the range of 190–260 nm and comparing them to the G6PD WT (Figure 5). Human G6PD is an α-helical protein, composed of β+α domain and a coenzyme binding domain with a classic β-α-β dinucleotide-binding fold. Consequently, the G6PD protein showed negative absorption bands at 208 and 222 nm, which are the characteristics of *α*-Helical protein. G6PD Murcia Oristano was the only G6PD variant with an absorption intensity similar to that of WT G6PD, but it had a slight difference in the intensity of its *β* absorption (a positive band between 195 and 200 nm). Each of the G6PD variants exhibited an absorption pattern similar to that of G6PD WT, but with varying intensities. A change in absorption intensity indicates an alteration in the flexibility or rigidity of the secondary structure. Overall, the mutations studied here had no effect on the G6PD protein secondary structure of the G6PD variants, they altered only the flexibility of the secondary structure.

##### 3.3.2.2 Thermal stability analysis

**FIGURE 6:**
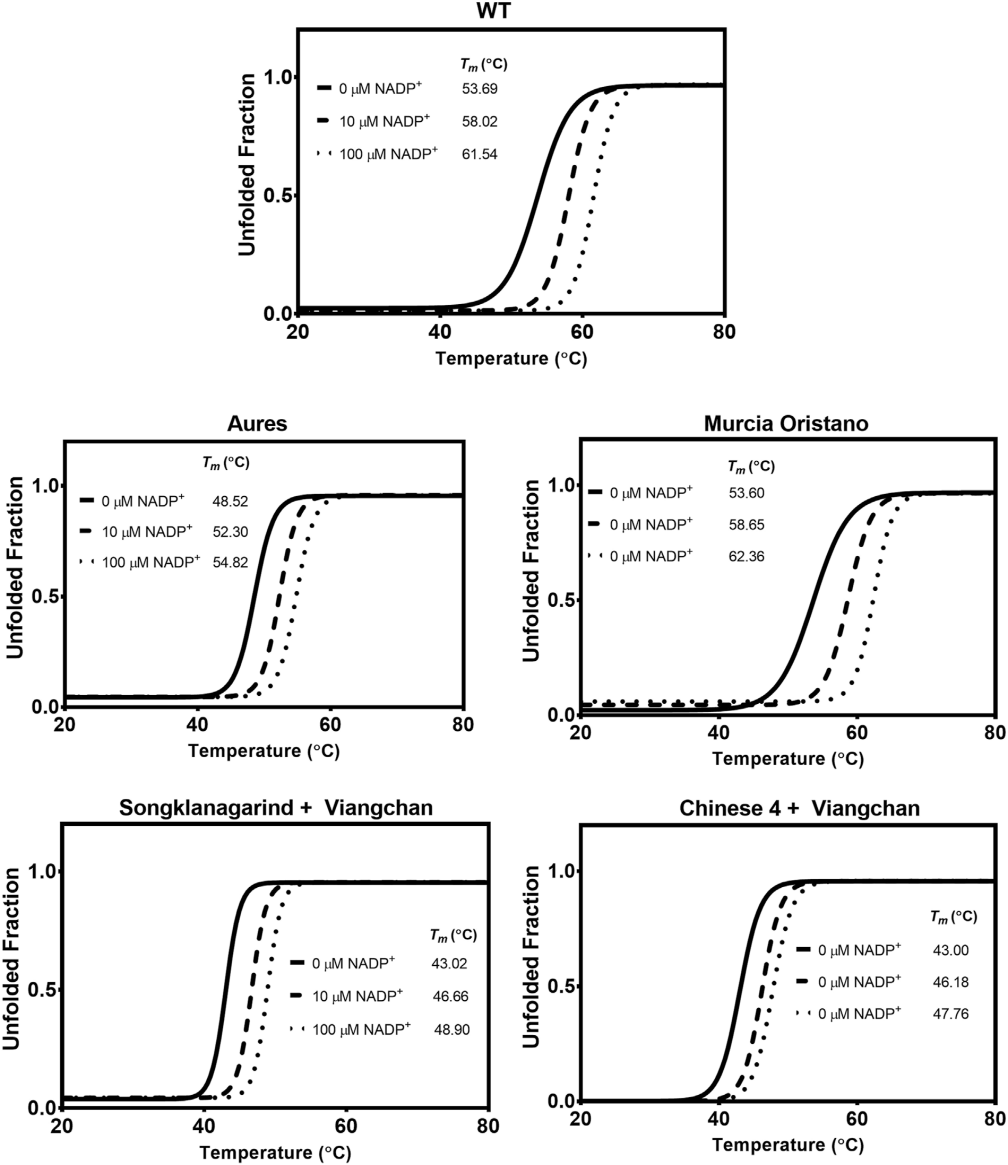
Thermal stability analysis of G6PD variants. In a Light- Cycler 480 real-time PCR machine, the reaction mixtures were heated at temperatures ranging from 20°C to 80°C, with excitation and emission wavelengths of 465 and 580 nm, respectively. The melting temperature (*T*_*m*_) was calculated and defined as the temperature at which half of the protein unfolded.

The thermostability of G6PD variants was then evaluated to determine how the mutations affected the G6PD structural stability as temperature increased. The *T*_*m*_ of each G6PD variant was determined and compared with that of the WT enzyme (Figure 6). Except for G6PD Murcia Oristano, the G6PD variants were less thermally stable than the WT enzyme. While the structural stability of G6PD Murcia Oristano, in the absence or presence of NADP^+^, was comparable to that observed for G6PD WT, the *T*_*m*_ values of G6PD Aures were approximately 5°C–6°C lower, indicating lower structural stability. The thermostability of the double mutants (G6PD Songklanagarind + Viangchan and G6PD Chinese 4 + Viangchan) was significantly lower than that of the G6PD WT, with *T*_*m*_ values of approximately 11°C–14°C lower. The structural NADP^+^ in the human G6PD protein structure is important for protein stability. In agreement with that, the presence of 10 and 100 µM NADP^+^ increased the *T*_*m*_ values of all G6PD proteins, improving protein thermostability.

##### 3.3.2.3 Structural stability of glucose-6-phosphate dehydrogenase variants upon denaturation by guanidine hydrochloride

**FIGURE 7:**
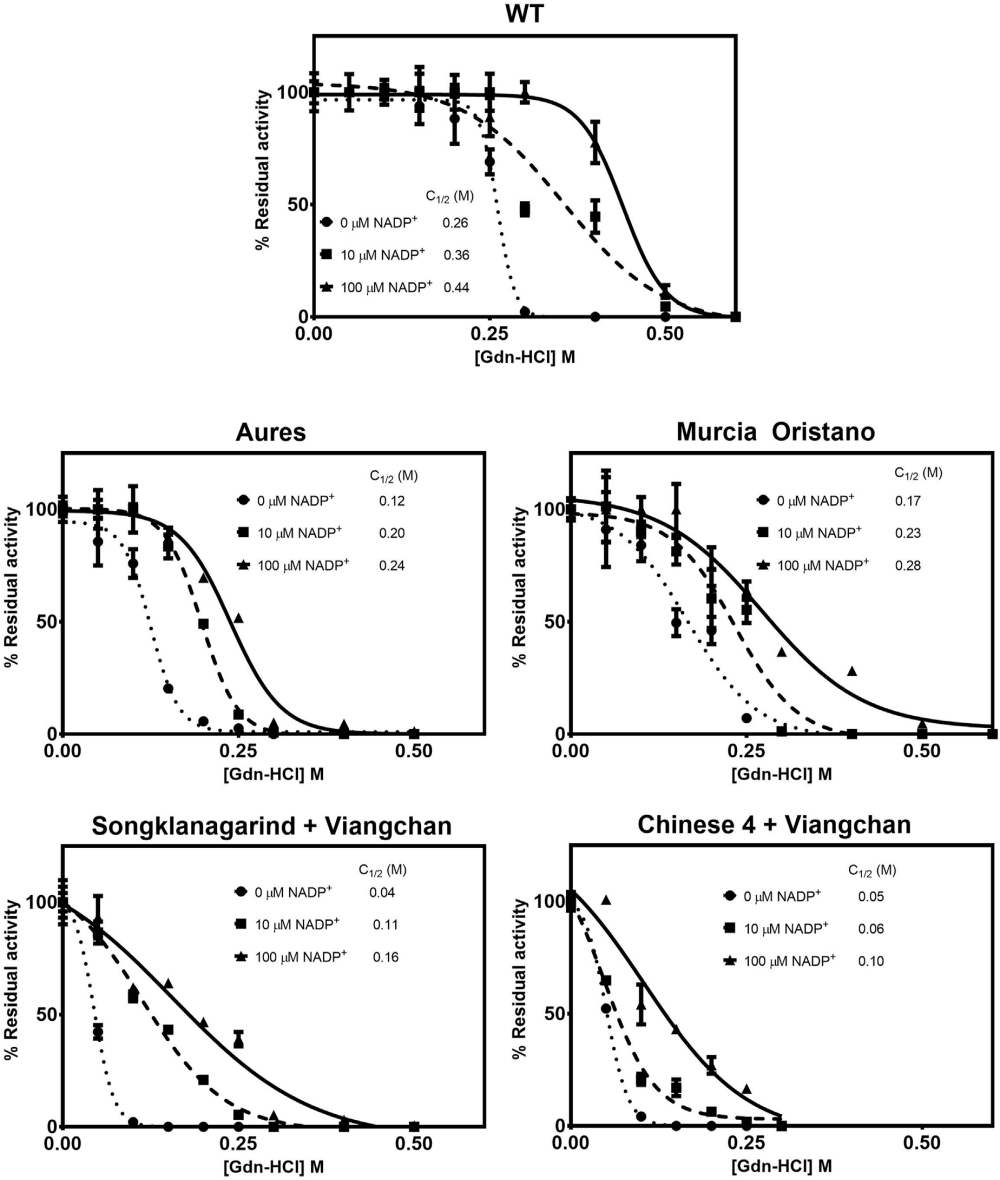
Susceptibility of G6PD variants to denaturation by Gdn-HCl. The protein was treated with different concentrations of Gdn-HCl (0–0.5 M) in the presence of various concentrations of NADP^+^ (0, 10, and 100 μM) at 37°C for 2 h. The residual enzyme activity was calculated as a percentage of the activity of the same enzyme incubated in the absence of Gdn-HCl.

Protein unfolding upon treatment with chemical denaturant can be used to assess structural stability. G6PD variants were exposed to varying concentrations of the chemical denaturant Gdn-HCl, and the concentration at which the protein lost half of its activity was defined as C_1/2_, with a higher C_1/2_ value indicating greater resistance to denaturation and thus greater structural stability. Under all tested conditions (in the absence and presence of 10 and 100 µM NADP^+^), all G6PD variants were structurally less stable than the G6PD WT, with double G6PD mutations having a greater effect on structural stability than the single G6PD mutations, as evidenced by lower C_1/2_ values (Figure 7). In agreement with the thermal stability analysis, the G6PD variant with the least effect on structural stability was G6PD Murcia Oristano, followed by G6PD Aures and G6PD Songklanagarind + Viangchan; G6PD Chinese 4 + Viangchan was the least stable G6PD mutant studied here. Both G6PD Songklanagarind + Viangchan and G6PD Chinese 4 + Viangchan had similar C_1/2_ values (0.04 and 0.05 M, respectively) in the absence of NADP^+^, whereas the presence of NADP^+^ appeared to more strongly improve the structural stability of G6PD Songklanagarind + Viangchan than that of G6PD Chinese 4 + Viangchan. The C_1/2_ values of Songklanagarind + Viangchan and Chinese 4 + Viangchan were 0.11 and 0.06 M, respectively, in the presence of 10 µM NADP^+^, and 0.16 and 0.10 M, respectively, in the presence of 100 M NADP^+^.

##### 3.3.2.4 Susceptibility of glucose-6-phosphate dehydrogenase variants to trypsin digestion

**FIGURE 8:**
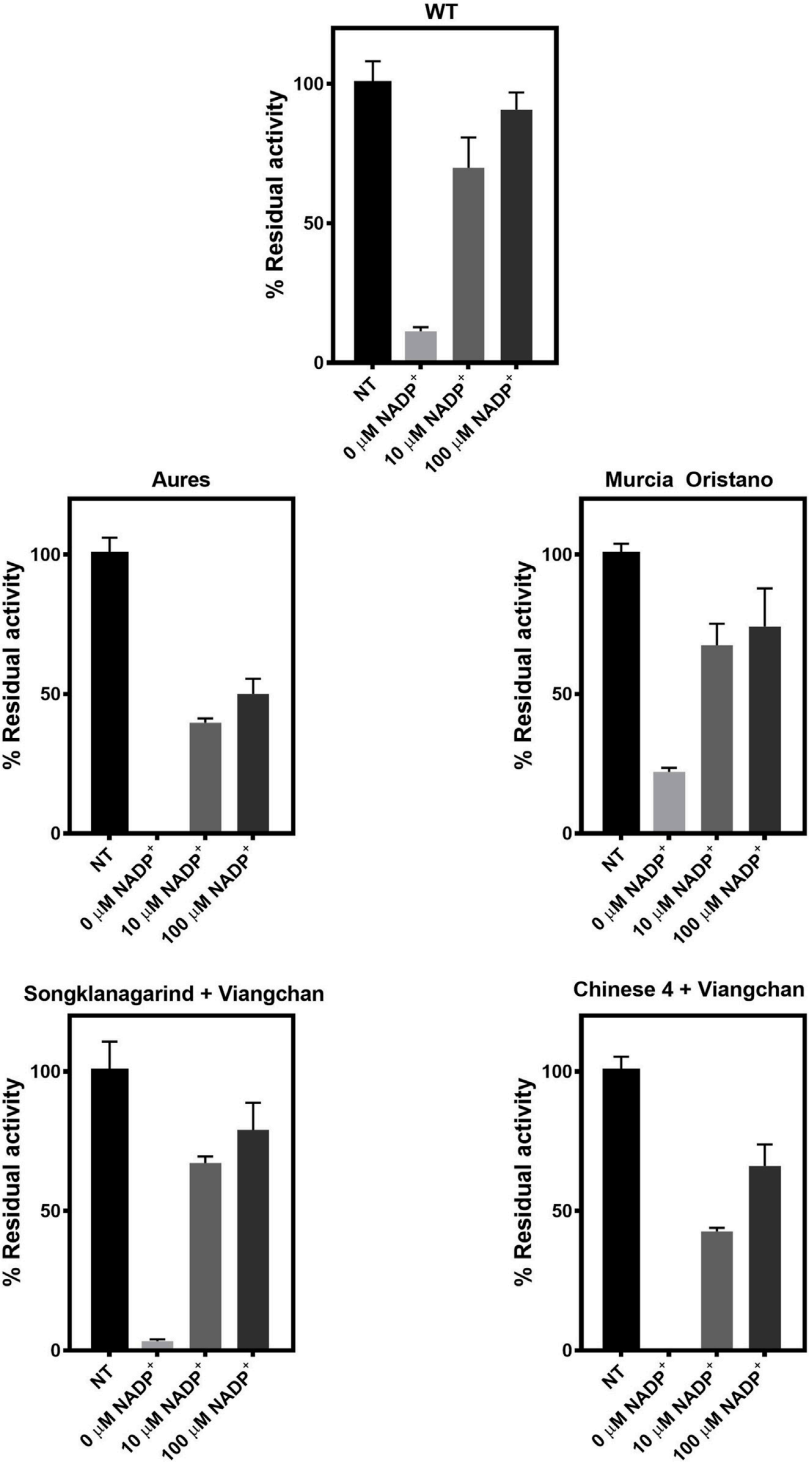
Susceptibility of G6PD variants to trypsin digestion. The protein was treated with trypsin (0.5 mg/ml) for 5 min at 25°C in the presence of various concentrations of NADP^+^ (0, 10, and 100 μM). The residual enzyme activity was calculated as a percentage of the activity of the same enzyme incubated in the absence of trypsin.

To further evaluate the structural stability of G6PD variants, the enzymes were treated with trypsin, and the residual enzyme activity was measured (Figure 8). In the absence of NADP^+^, all tested G6PD proteins lost 80%–100% of their activity, with G6PD Murcia Oristano (22%) retaining more residual activity than the WT enzyme (11%). G6PD Songklanagarind + Viangchan retained 3% of its activity, whereas G6PD Aures and G6PD Chinese 4 + Viangchan completely lost their activities, indicating that these G6PD variants had the lowest protein stability. At both tested concentrations (10 and 100 µM), NADP^+^ was found to stabilize protein structure by increasing the residual enzyme activity of all G6PD proteins. The stabilizing effect of NADP^+^ was the most pronounced in G6PD Songklanagarind + Viangchan, where the presence of 10 and 100 µM of NADP^+^ retained 64% and 76%, respectively, of G6PD activity.

## 4 Discussion

The risk of hemolysis in people with G6PD deficiency has restricted the use of 8-aminoquinoline for the radical cure of vivax malaria. To identify those who are eligible for radical treatment with 8-aminoquinolines, it is critical to assess the hemolytic risk, which can be done by determining the prevalence and genotype of G6PD deficiency. Treatment with primaquine is recommended at a dose of 15 mg/day for 14 days in G6PD-normal individuals and at a dose of 0.75 mg/kg for 8 weeks in G6PD-deficient persons (Organization, 2016a). In contrast, tafenoquine is prescribed as a single 300-mg dose, which poses a greater risk of hemolysis; consequently, this drug is prescribed only to those who demonstrate at least 70% G6PD enzyme activity on a quantitative test (Llanos-Cuentas et al., 2019). However, routine G6PD testing is not available in resource-constrained settings. Additionally, most G6PD testing is done with qualitative tests that are incapable of identifying heterozygous female individuals.

Malaria hotspots are located along the western region of Thailand where *P. vivax* has been responsible for more than 90% of total malaria cases, necessitating the use of 8-aminoquinolines for radical cure (Malaria situation in Thailand, 2022). Previous studies conducted using phenotypic tests reported that the prevalence of G6PD deficiency ranged between 7% and 14% among people living along the Thai‒Myanmar border (Bancone et al., 2017a; Bancone et al., 2017b; Boonyuen et al., 2021). Here, we report a prevalence of G6PD deficiency of 6.76% among 1,125 people in western Thailand, as determined using a quantitative phenotypic test based on WST-8. Because female individuals with heterozygous G6PD deficiency typically exhibit a wide range of G6PD activity levels owing to random X-inactivation, phenotypic testing alone is ineffective at identifying them. It was reported that a female heterozygous for G6PD Mahidol was misclassified as G6PD normal, resulting in hemolysis when she was treated with primaquine (Chu et al., 2017). Thus, a genotypic test is required to confirm the G6PD status in those suspected of having G6PD deficiency. We improved on our previous HRM assays, expanding the tests to detect 12 G6PD mutations (common in Southeast Asia) over three reactions. The assays were 100% sensitive and 100% specific, as compared with direct DNA sequencing. Multiplexed HRM assays revealed a G6PD deficiency frequency of 22.40% (252/1,125) in the studied population, with G6PD Mahidol being the most common variant, accounting for 99.60% of the detected mutations. Overall, 77 and 174 G6PD Mahidol cases were observed in male and female participants, respectively, as well as one G6PD Aures case in a female participant. The findings of this study complement previous findings in Thai‒Myanmar border populations that G6PD Mahidol was the most common variant among Karen and Burmese people (Nuchprayoon et al., 2008; Bancone et al., 2014; Boonyuen et al., 2021). The phenotype-genotype association analysis revealed a wide range of G6PD activity levels in those carrying G6PD Mahidol, especially in female individuals. The level of G6PD activity was found to be greater than 30% in nine male and 167 female individuals with G6PD Mahidol. Furthermore, the average G6PD activity level of female participants carrying G6PD Mahidol was comparable to that of G6PD-normal female individuals. Thus, our findings indicate that phenotypic testing alone may not be enough to detect G6PD deficiency, even in hemizygous male individuals. Similar incidents have previously been reported in which individuals carrying the Mahidol mutation were classified as G6PD normal by phenotypic tests (Chu et al., 2017; Chu et al., 2019). People carrying other G6PD variants (G6PD Santa Maria, G6PD A^−^, G6PD Viangchan, G6PD Gaohe, G6PD Chinese 4, G6PD Chinese 5, G6PD Canton and G6PD Kaiping) were also found to have enzyme activity levels of greater than 30% (Powers et al., 2018; He et al., 2020; Boonyuen et al., 2021). Importantly, a misclassification of G6PD status may increase the hemolytic risk of radical treatment with 8-aminoquinolines. Despite the fact that G6PD Mahidol is a mild enzyme deficiency, hemolysis events have been reported in people receiving primaquine or tafenoquine (Chu et al., 2017; Rueangweerayut et al., 2017). Female individuals heterozygous for G6PD Mahidol (with an enzyme activity of 80%) still experienced hemolysis when given 300 mg tafenoquine. Though the catalytic activity of G6PD was only mildly affected by the Mahidol mutation, biochemical analysis revealed that this mutation caused protein instability, resulting in dimer dissociation and enzyme deficiency (Boonyuen et al., 2016).

G6PD Aures was identified by multiplexed HRM assays in a heterozygous female participant with a G6PD activity level of 11.2 U/gHb, which is greater than the 30% cut-off. The Aures mutation is common in Arab countries, but it was first reported in Thailand in 2018 and is common among Lao Theung people (Bayoumi et al., 1996; Alfadhli et al., 2005; Sanephonasa et al., 2021). Concerns about hemolysis in this group should be raised, as *P. vivax* is responsible for nearly 20% of malaria cases in the Arab world (Organization, 2021). G6PD Aures, like G6PD Mahidol, is classified as a class III variant with mild enzyme deficiency. According to our findings, the G6PD Aures mutation reduced catalytic activity by 2.5‒fold while having a moderate effect on protein structural stability, similar to the observed effects in G6PD Mahidol but with lower catalytic efficiency. As a result, it is possible that similar incidence of hemolysis may occur in those who carry G6PD Aures. Thus, G6PD quantitative tests should be used in the Arab world and in other areas where G6PD Aures is common to reduce the risk of hemolysis, and G6PD genotyping may be required in female individuals suspected of having G6PD deficiency.

The phenotypic test revealed two samples with deficient G6PD activity but no mutations detected by our HRM assays. G6PD Kaiping, which was not included in our assays, was discovered *via* DNA sequencing in these two samples. Although our multiplexed HRM assays can correctly identify 12 G6PD variants, they are unable to detect the zygosity of the samples and may not be suitable for G6PD genotyping in other regions owing to the variable prevalence of G6PD mutations. With simple design and easy interpretation, our HRM assays can be optimized, and the desired G6PD mutations can be included to enable G6PD genotyping in other areas. In fact, previous studies using different designs and more complicated result analyses demonstrated that HRM assays can detect zygosity in samples (Yan et al., 2010; Islam et al., 2018); however, those tests could detect only 1‒2 mutations in a single reaction, making them impractical for use with large sample sizes. The currently available G6PD genotypic platforms appear to have similar limited applications; they are either expensive, time-consuming, or complicated. Because most genotypic tests were designed to detect only common mutations in specific regions, they are incapable of detecting rare mutations.

Through DNA sequencing, we discovered G6PD Murcia Oristano and the double mutant G6PD Songklanagarind + Viangchan for the first time in Thailand from validated samples. HRM assays were also able to identify another double mutant, G6PD Chinese 4 + Viangchan. G6PD Murcia Oristano was previously reported among Spanish people and was classified as class III variant (Rovira et al., 1995). In this study, G6PD Murcia Oristano was detected in a family with two sons showing deficient G6PD activity and a mother with intermediate enzyme activity. The biochemical and structural properties of G6PD Murcia Oristano were comparable to those of G6PD Mahidol but with a higher *K*_m_ for the NADP^+^ substrate. The Tyr70Cys mutation in G6PD Murcia Oristano slightly altered the flexibility of the secondary structure and the protein structural stability. The Murcia Oristano mutation appeared to have a milder effect compared with the Aures and Mahidol mutations, implying a lower risk of hemolysis. However, more clinical data are required to confirm this assumption.

Multiple mutations were previously shown to have a greater effect on G6PD deficiency than single mutations (Nantakomol et al., 2013). The combined effects of two or more mutations on catalytic activity and structural stability were observed (Praoparotai et al., 2020; Pakparnich et al., 2021). Here, G6PD Songklanagarind + Viangchan and G6PD Chinese 4 + Viangchan variants were observed in female individuals with extremely low enzyme activity. Biochemical characterization indicated that both variants had a significant effect on enzyme activity and protein stability, which is consistent with previous findings. These G6PD variants were 20‒fold less catalytically active compared with the WT enzyme, and the presence of double mutation markedly reduced structural stability upon exposure to heat, denaturant, or trypsin. G6PD Songklanagarind and G6PD Viangchan are class II variants, whereas G6PD Chinese 4 is a mildly deficient class III variant. However, regardless of the single variant classification, combinations of class II (Songklanagarind and Viangchan) and class II and III (G6PD Chinese 4 + Viangchan) variants produced individuals with severe enzyme deficiency. Multiple mutations were found mostly in female participants with severe enzyme deficiency, implying that routine phenotypic tests should be able to detect them. Notably, the risk of hemolysis is higher in individuals with the multiple G6PD mutations.

Determining the safety of radical treatment with 8-aminoquinolines is an important issue before drug administration, particularly in areas where G6PD deficiency is common. Drug administration without G6PD testing has caused medical complications in the management of hemolysis, which may have a negative impact on the patient as well as inciting public concern (Chu et al., 2019). When G6PD testing is not available, it is frequently decided not to prescribe the drugs, despite their effectiveness in killing hypnozoites and preventing relapse, causing a significant burden for vivax malaria eradication. It has become more difficult to prescribe 8-aminoquinolines, particularly tafenoquine because this drug is prescribed as a 300-mg dose, which can cause hemolysis and cannot be stopped during the drug course, unlike primaquine. As a result, attempts have been made to reduce the risk of hemolysis by lowering the drug dose, which has led to another issue of adherence (Alving et al., 1960). Furthermore, low-dose prescription may lead to a high recurrence rate of *P. vivax* (John et al., 2012). Therefore, eliminating vivax malaria remains a significant challenge that necessitates additional efforts in vivax endemic areas to provide evidence on safe and effective anti-relapse therapy using 8-aminoquinolines.

## 5 Conclusion

Information on the prevalence of G6PD deficiency is useful to estimate those who are eligible to receive 8-aminoquinolines for radical treatment. With a sample size of 1,125 people, our findings suggest that up to 22% of people in western Thailand may be ineligible. Furthermore, people with the Mahidol mutation had G6PD activity that was greater than the 30% cut-off, and heterozygous female individuals had G6PD activity that was comparable to that of G6PD-normal persons. This meant that routine qualitative tests could misclassify these individuals as G6PD normal, putting them at a significant hemolytic risk when 8-aminoquinolines are prescribed. In regions where class III variants with mild enzyme deficiency are common, our findings suggest that the G6PD status should be assessed quantitatively, especially before the administration of tafenoquine. Furthermore, G6PD genotyping should be used in conjunction with hemolytic risk assessment in those suspected of having G6PD deficiency, particularly in female patients. Individuals with double mutations are more likely to have hemolysis than are those with single mutations, according to our biochemical data, because the double mutations significantly reduce catalytic activity as well as the structural stability of the G6PD protein.

## Data Availability

The original contributions presented in the study are included in the article/Supplementary Material, further inquiries can be directed to the corresponding author.
