# Supplementary material for: Genotype-phenotype association and biochemical analyses of glucose-6-phosphate dehydrogenase variants: Implications for the hemolytic risk of using 8-aminoquinolines for radical cure
Source: Front Pharmacol. 2022 Oct 20;13:1032938. doi: 10.3389/fphar.2022.1032938 (PMC9631214; doi:10.3389/fphar.2022.1032938)
Supplement: Supplementary file 1 [file DataSheet1.PDF]

Supplementary data

Genotype-phenotype association and biochemical analyses of glucose-6-phosphate dehydrogenase (G6PD) variants: implications for the hemolytic risk of using 8-aminoquinolines for radical cure

Sirapapha Sudsumrit1, Kamonwan Chamchoy2, Duantida Songdej3, Poom Adisakwattana4, Srivicha Krudsood5, Emily R Adams6, Mallika Imwong1, Ubolsree Leartsakulpanich7, Usa Boonyuen1,*

[1]Department of Molecular Tropical Medicine and Genetics, Faculty of Tropical Medicine, Mahidol University, Bangkok 10400 Thailand

[2]Princess Srisavangavadhana College of Medicine, Chulabhorn Royal Academy, Bangkok 10210, Thailand

[3]Department of Pediatrics, Faculty of Medicine Ramathibodi Hospital, Mahidol University, Bangkok 10400 Thailand

[4]Department of Helminthology, Faculty of Tropical Medicine, Mahidol University, Bangkok 10400 Thailand

[5]Department of Tropical Hygiene, Faculty of Tropical Medicine, Mahidol University, Bangkok 10400 Thailand

[6]Research Centre for Drugs and Diagnostics, Liverpool School of Tropical Medicine, Liverpool, UK

[7]National Center for Genetic Engineering and Biotechnology, National Science and Technology Development Agency, Pathumthani, 12120, Thailand

*Corresponding author: Usa Boonyuen, Department of Molecular Tropical Medicine and Genetics, Faculty of Tropical Medicine, Mahidol University, Bangkok 10400 Thailand

Email address: usa.boo@mahidol.ac.th, usa.boo@mahidol.edu

Table S1. Purification of recombinant G6PD variants.

Construct	Purification step	Total protein (mg)	Total activity (IU)	Yield (%)
WT	Crude extract	210	2,500	100
	Affinity chromatography	25	1,500	60
Aures	Crude extract	190	1,650	100
	Affinity chromatography	20	950	58
Murcia Oristano	Crude extract	215	2,150	100
	Affinity chromatography	23	1,150	53
Songklanagarind + Viangchan	Crude extract	185	760	100
	Affinity chromatography	12	175	23
Chinese 4 + Viangchan	Crude extract	190	800	100
	Affinity chromatography	15	180	23

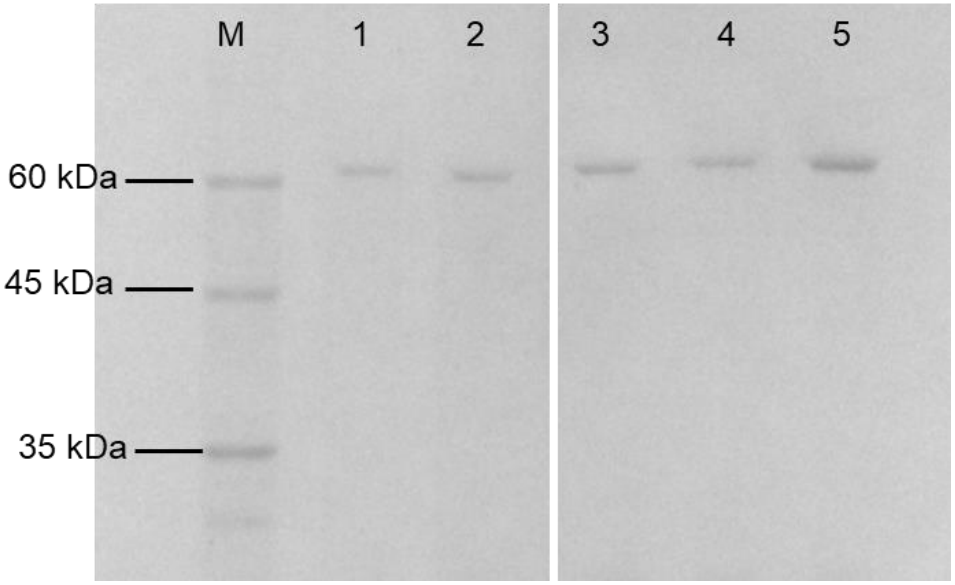

Figure S1. SDS-PAGE analysis of purified recombinant G6PD variants: lane M, molecular mass marker protein; lane 1, G6PD WT; lane 2, G6PD Aures; lane 3, G6PD Murcia Oristano; lane 4, G6PD Chinese 4 + Viangchan and lane 5, G6PD Songklanagarind + Viangchan.
